# Disodium diaqua­bis­(methyl­enedi­phos­pho­nato-κ^2^ *O*,*O*′)cobaltate(II) dihydrate

**DOI:** 10.1107/S1600536811038530

**Published:** 2011-09-30

**Authors:** Kina van Merwe, Hendrik G. Visser, Johan A. Venter

**Affiliations:** a Department of Chemistry, University of the Free State, PO Box 339, Bloemfontein, 9330, South Africa

## Abstract

In the title compound, Na_2_[Co(CH_4_O_6_P_2_)_2_(H_2_O)_2_]·2H_2_O, the asymmetric unit is composed of one methyl­enediphospho­nate ligand and one water mol­ecule, which both are coordinated to a Co^II^ atom, as well as a non-coordinated water mol­ecule and a sodium cation. The Co^II^ atom occupies a special position on a crystallographic inversion centre. The slightly distorted Co^II^O_6_ octa­hedral coordination environment is composed of two bidentate methyl­enediphospho­nate ligands and two coordinated water mol­ecules in *trans* positions. The sodium ion is octa­hedrally coordinated to six O atoms with Na—O distances ranging from 2.3149 (12) to 2.6243 (12) Å. An extensive three-dimensional network of inter­molecular as well as intra­molecular O—H⋯O and C—H⋯O hydrogen bonding inter­acions is present.

## Related literature

For general background to organic diphospho­nic acids, see: Vega *et al.* (1996 ▶). For related structures, see: Bon *et al.* (2010 ▶); DeLaMatter *et al.* (1973 ▶); Harmony *et al.* (1984 ▶); Jurisson *et al.* (1983 ▶); Van der Merwe *et al.* (2010 ▶). For bond lengths and angles in related structures, see: Bao *et al.* (2003 ▶); Cao *et al.* (2007 ▶); Gong *et al.* (2006 ▶); Van der Merwe *et al.* (2009 ▶); Visser *et al.* (2010 ▶); Yin *et al.* (2003 ▶).
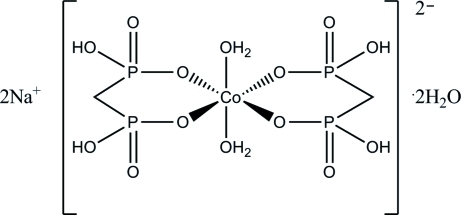


## Experimental

### 

#### Crystal data

Na_2_[Co(CH_4_O_6_P_2_)_2_(H_2_O)_2_]·2H_2_O
*M* *_r_* = 524.94
Monoclinic, 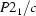

*a* = 6.8694 (2) Å
*b* = 13.2860 (4) Å
*c* = 8.3541 (3) Å
β = 91.375 (1)°
*V* = 762.23 (4) Å^3^
*Z* = 2
Mo *K*α radiation
μ = 1.69 mm^−1^
*T* = 100 K
0.52 × 0.24 × 0.09 mm

#### Data collection

Bruker APEXII CCD diffractometer
Absorption correction: multi-scan (*SADABS*, Bruker, 2007 ▶) *T*_min_ = 0.474, *T*_max_ = 0.863
8742 measured reflections
1901 independent reflections
1838 reflections with *I* > 2σ(*I*)
*R*_int_ = 0.022

#### Refinement

*R*[*F* ^2^ > 2σ(*F* ^2^)] = 0.019
*wR*(*F* ^2^) = 0.055
*S* = 1.13
1901 reflections
146 parameters
7 restraints
All H-atom parameters refined
Δρ_max_ = 0.36 e Å^−3^
Δρ_min_ = −0.57 e Å^−3^

### 

Data collection: *APEX2* (Bruker, 2007 ▶); cell refinement: *SAINT-Plus* (Bruker, 2007 ▶); data reduction: *SAINT-Plus*; program(s) used to solve structure: *SHELXS97* (Sheldrick, 2008 ▶); program(s) used to refine structure: *SHELXL97* (Sheldrick, 2008 ▶); molecular graphics: *DIAMOND* (Brandenburg & Putz, 2005 ▶); software used to prepare material for publication: *WinGX* (Farrugia, 1999 ▶).

## Supplementary Material

Crystal structure: contains datablock(s) global, I. DOI: 10.1107/S1600536811038530/wm2520sup1.cif

Structure factors: contains datablock(s) I. DOI: 10.1107/S1600536811038530/wm2520Isup2.hkl

Additional supplementary materials:  crystallographic information; 3D view; checkCIF report

## Figures and Tables

**Table 1:** Selected bond lengths (Å)

Co1—O1	2.0886 (10)
Co1—O7	2.0900 (10)
Co1—O2^i^	2.1141 (10)

Symmetry code: (i) 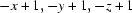.

**Table 2:** Hydrogen-bond geometry (Å, °)

*D*—H⋯*A*	*D*—H	H⋯*A*	*D*⋯*A*	*D*—H⋯*A*
O8—H6⋯O7	0.86 (1)	1.88 (1)	2.7154 (15)	164 (2)
C1—H4⋯O1^i^	0.92 (2)	2.54 (2)	3.1449 (17)	123.9 (15)
C1—H3⋯O4^ii^	0.92 (2)	2.53 (2)	3.4366 (17)	168.4 (17)
O3—H2⋯O2^ii^	0.81 (3)	1.84 (3)	2.6394 (14)	176 (3)
O1—H1*A*⋯O6^iii^	0.83 (2)	1.98 (2)	2.8008 (14)	173 (2)
O8—H7⋯O1^iii^	0.83 (1)	2.57 (2)	3.2763 (15)	143 (2)
O1—H1*B*⋯O4^iv^	0.83 (2)	1.84 (2)	2.6634 (15)	175 (2)
O5—H5⋯O6^v^	0.82 (2)	1.81 (2)	2.6272 (14)	177 (3)

Symmetry codes: (i) 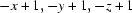; (ii) 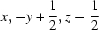; (iii) 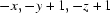; (iv) 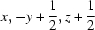; (v) 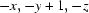.

**Fig. 1.**
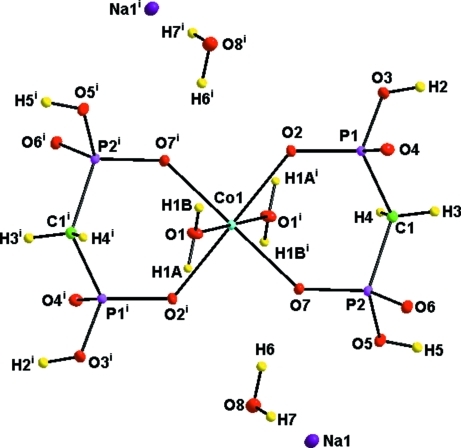
Representation of the title compound, showing the numbering scheme and displacement ellipsoids drawn at the 50% probability level. [Symmetry code: (i) 1-x, 1-y, -z].

**Fig. 2.**
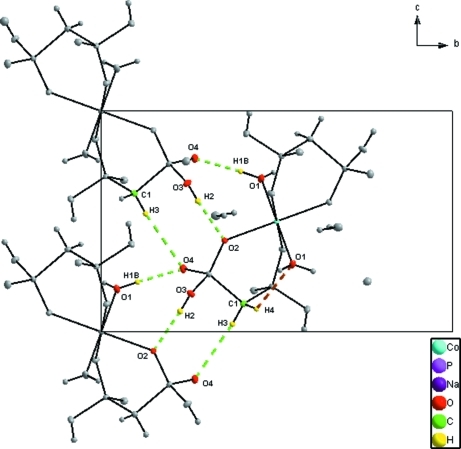
The crystal packing of the title compound viewed along the a axis. Green dashed lines are employed to represent intermolecular hydrogen bonding and an orange dashed line was used to displace the intramolecular hydrogen bonding.

**Fig. 3.**
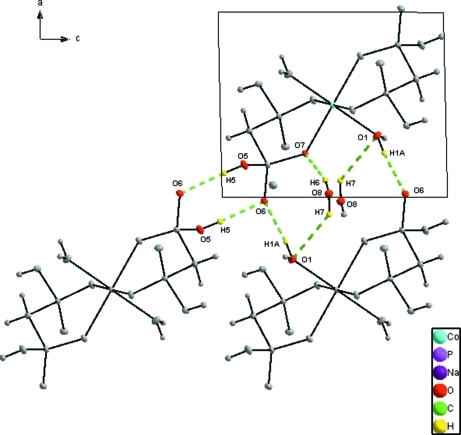
The packing diagram of the title compound viewed along the b axis. Green dashed lines are utilized to display the extensive intermolecular hydrogen bonding.

## supplementary crystallographic information

### Comment

The title compound forms part of an ongoing study involving methylene diphosphonate, and its coordination to various metal cores.

In the past 20 years numerous diphosphonate compounds have undergone intensive pharmacological studies primarily because of their possible use in treating bone diseases. This can be attributed to the fact that bisphosphonic acids are excellent anti-hypercalcemics and have a high affinity for bone tissue (Vega *et al.*, 1996).

In the title compound, Na_2_[Co(CH_4_O_6_P_2_)_2_(H_2_O)_2_]^.^2H_2_O, (Fig. 1, Table 1), the asymmetric unit is composed of one methylene diphosphonate ligand and one aqueous molecule which are coordinated to a Co(II) atom, as well as a non-coordinated aqueous solvent molecule and a sodium cation. The Co(II) atom occupies a special position on a crystallographic inversion centre. The sodium ion is octahedrally coordinated to six oxygen atoms with Na—O distances ranging from 2.3149 (12) to 2.6243 (12) Å. The octahedral geometry around the Co^II^ metal center is slightly distorted with O—Co—O angles ranging between 86.63 (4) ° and 93.14 (4) °. The Co—O bond lengths vary between 2.0886 (10) and 2.1141 (10) Å. These distances correspond to literature values (Bao *et al.* (2003); Cao *et al.* (2007); Gong *et al.* (2006); Yin *et al.* (2003); Van der Merwe *et al.* (2009); Visser *et al.* (2010).

A three-dimensional network is provided by numerous C—H–O and O—H–O hydrogen bonds (Figs. 2, 3 and Table 2).

### Experimental

CoCl_2_^.^6H_2_O (0,1696 g, 0,00071 mol) and methylene diphosphonate (0,3726 g, 0,00212 mol) were dissolved in distilled water (5 cm^3^). Sodium hydroxide (3 cm^3^, 1 *M*) was gradually added to the solution mixture which was heated for three days at 413 K. The final pH of the solution was adjusted to 1.23 with hydrochloric acid. Pink crystals, suitable for X-ray diffraction, were obtained. (Yield: 27.6%)

### Refinement

All H atoms were located from difference Fourier maps and were refined isotropically without further restraints. The highest residual electron density was located 0.78 Å from C1.

### Crystal data

**Table:**

Na_2_[Co(CH_4_O_6_P_2_)_2_(H_2_O)_2_]·2H_2_O	*F*(000) = 530
*M**_r_* = 524.94	*D*_x_ = 2.287 Mg m^−^^3^
Monoclinic, *P*2_1_/*c*	Mo *K*α radiation, λ = 0.71073 Å
Hall symbol: -P 2ybc	Cell parameters from 6202 reflections
*a* = 6.8694 (2) Å	θ = 2.9–28.4°
*b* = 13.2860 (4) Å	µ = 1.69 mm^−^^1^
*c* = 8.3541 (3) Å	*T* = 100 K
β = 91.375 (1)°	Plate, pink
*V* = 762.23 (4) Å^3^	0.52 × 0.24 × 0.09 mm
*Z* = 2	

### Data collection

**Table:**

Bruker APEXII CCD diffractometer	1838 reflections with *I* > 2σ(*I*)
phi and ω scans	*R*_int_ = 0.022
Absorption correction: multi-scan (*SADABS*, Bruker, 2007)	θ_max_ = 28.4°, θ_min_ = 3.1°
*T*_min_ = 0.474, *T*_max_ = 0.863	*h* = −8→9
8742 measured reflections	*k* = −17→16
1901 independent reflections	*l* = −11→8

### Refinement

**Table:**

Refinement on *F*^2^	7 restraints
Least-squares matrix: full	All H-atom parameters refined
*R*[*F*^2^ > 2σ(*F*^2^)] = 0.019	*w* = 1/[σ^2^(*F*_o_^2^) + (0.0289*P*)^2^ + 0.4299*P*] where *P* = (*F*_o_^2^ + 2*F*_c_^2^)/3
*wR*(*F*^2^) = 0.055	(Δ/σ)_max_ = 0.001
*S* = 1.13	Δρ_max_ = 0.36 e Å^−^^3^
1901 reflections	Δρ_min_ = −0.57 e Å^−^^3^
146 parameters	

### Special details

**Table:**

Geometry. All s.u.'s (except the s.u. in the dihedral angle between two l.s. planes) are estimated using the full covariance matrix. The cell s.u.'s are taken into account individually in the estimation of s.u.'s in distances, angles and torsion angles; correlations between s.u.'s in cell parameters are only used when they are defined by crystal symmetry. An approximate (isotropic) treatment of cell s.u.'s is used for estimating s.u.'s involving l.s. planes.

### Fractional atomic coordinates and isotropic or equivalent isotropic displacement parameters (Å^2^)

**Table:**

	*x*	*y*	*z*	*U*_iso_*/*U*_eq_	
Co1	0.5	0.5	0.5	0.00488 (8)	
P1	0.43774 (5)	0.30632 (3)	0.26142 (4)	0.00528 (9)	
P2	0.17626 (5)	0.48657 (3)	0.20454 (4)	0.00578 (9)	
Na1	0.06611 (8)	0.75775 (4)	0.22722 (7)	0.01006 (13)	
O1	0.33424 (15)	0.45566 (8)	0.69412 (12)	0.0092 (2)	
O2	0.52678 (14)	0.35119 (7)	0.41366 (11)	0.00758 (19)	
O3	0.61219 (15)	0.25484 (8)	0.17312 (12)	0.0084 (2)	
O4	0.27445 (14)	0.23364 (8)	0.28565 (12)	0.0087 (2)	
O5	0.17550 (15)	0.58645 (8)	0.10142 (12)	0.0091 (2)	
O6	−0.02026 (14)	0.43517 (8)	0.18700 (11)	0.00828 (19)	
O7	0.23609 (15)	0.51625 (8)	0.37290 (12)	0.0081 (2)	
O8	0.02069 (15)	0.67436 (8)	0.47388 (12)	0.0114 (2)	
C1	0.3604 (2)	0.40539 (10)	0.12696 (15)	0.0072 (2)	
H1A	0.241 (3)	0.4908 (15)	0.722 (3)	0.025 (6)*	
H1B	0.321 (3)	0.3969 (13)	0.726 (3)	0.029 (6)*	
H2	0.582 (4)	0.225 (2)	0.093 (3)	0.037 (7)*	
H3	0.320 (3)	0.3732 (15)	0.035 (2)	0.015 (5)*	
H4	0.469 (3)	0.4420 (15)	0.103 (2)	0.012 (4)*	
H5	0.131 (4)	0.5780 (19)	0.011 (2)	0.038 (7)*	
H6	0.092 (3)	0.6221 (13)	0.461 (3)	0.035 (5)*	
H7	−0.091 (2)	0.6498 (18)	0.474 (3)	0.035 (5)*	

### Atomic displacement parameters (Å^2^)

**Table:**

	*U*^11^	*U*^22^	*U*^33^	*U*^12^	*U*^13^	*U*^23^
Co1	0.00514 (14)	0.00495 (14)	0.00451 (13)	−0.00021 (8)	−0.00063 (9)	−0.00021 (8)
P1	0.00519 (16)	0.00539 (17)	0.00524 (15)	0.00033 (12)	−0.00009 (11)	−0.00059 (11)
P2	0.00623 (17)	0.00582 (17)	0.00523 (17)	0.00061 (12)	−0.00118 (12)	−0.00022 (11)
Na1	0.0087 (3)	0.0113 (3)	0.0102 (3)	0.0003 (2)	−0.0008 (2)	0.0009 (2)
O1	0.0100 (5)	0.0072 (5)	0.0105 (4)	0.0011 (4)	0.0035 (4)	0.0019 (4)
O2	0.0107 (5)	0.0061 (5)	0.0059 (4)	0.0008 (4)	−0.0019 (3)	−0.0008 (3)
O3	0.0068 (5)	0.0104 (5)	0.0081 (4)	0.0017 (4)	−0.0002 (4)	−0.0032 (4)
O4	0.0072 (5)	0.0074 (5)	0.0116 (4)	−0.0008 (4)	0.0001 (3)	0.0000 (4)
O5	0.0121 (5)	0.0067 (5)	0.0084 (4)	0.0001 (4)	−0.0035 (4)	0.0013 (4)
O6	0.0071 (4)	0.0094 (5)	0.0083 (4)	−0.0004 (4)	−0.0013 (3)	−0.0002 (4)
O7	0.0080 (5)	0.0098 (5)	0.0064 (4)	0.0021 (4)	−0.0021 (3)	−0.0015 (3)
O8	0.0091 (5)	0.0141 (5)	0.0109 (5)	0.0018 (4)	0.0006 (4)	−0.0003 (4)
C1	0.0076 (6)	0.0077 (6)	0.0061 (6)	0.0001 (5)	0.0000 (5)	0.0000 (5)

### Geometric parameters (Å, °)

**Table:**

Co1—O1	2.0886 (10)	Na1—O3^iii^	2.3431 (12)
Co1—O1^i^	2.0886 (10)	Na1—O4^iv^	2.3612 (11)
Co1—O7	2.0900 (10)	Na1—O6^iv^	2.4860 (12)
Co1—O7	2.0900 (10)	Na1—O5	2.6243 (12)
Co1—O7^i^	2.0900 (10)	O1—H1A	0.831 (16)
Co1—O2	2.1141 (10)	O1—H1B	0.830 (16)
Co1—O2^i^	2.1141 (10)	O3—Na1^v^	2.3431 (12)
P1—O4	1.4975 (10)	O3—H2	0.81 (3)
P1—O2	1.5198 (10)	O4—Na1^vi^	2.3612 (11)
P1—O3	1.5781 (10)	O5—H5	0.815 (16)
P1—C1	1.8021 (14)	O6—Na1^vi^	2.4860 (12)
P2—O7	1.5083 (10)	O8—Na1^vii^	2.3149 (12)
P2—O7	1.5083 (10)	O8—H6	0.856 (13)
P2—O6	1.5169 (10)	O8—H7	0.834 (13)
P2—O5	1.5820 (10)	C1—H3	0.92 (2)
P2—C1	1.7954 (14)	C1—H4	0.92 (2)
Na1—O8^ii^	2.3149 (12)		
			
O1—Co1—O1^i^	180	O5—P2—C1	107.49 (6)
O1—Co1—O7	86.63 (4)	O8^ii^—Na1—O3^iii^	115.88 (4)
O1^i^—Co1—O7	93.37 (4)	O8^ii^—Na1—O4^iv^	84.24 (4)
O1—Co1—O7	86.63 (4)	O3^iii^—Na1—O4^iv^	159.88 (4)
O1^i^—Co1—O7	93.37 (4)	O8^ii^—Na1—O6^iv^	83.04 (4)
O1—Co1—O7^i^	93.37 (4)	O3^iii^—Na1—O6^iv^	92.19 (4)
O1^i^—Co1—O7^i^	86.63 (4)	O4^iv^—Na1—O6^iv^	90.55 (4)
O7—Co1—O7^i^	180	O8^ii^—Na1—O5	90.31 (4)
O7—Co1—O7^i^	180	O3^iii^—Na1—O5	81.45 (4)
O1—Co1—O2	93.14 (4)	O4^iv^—Na1—O5	99.03 (4)
O1^i^—Co1—O2	86.86 (4)	O6^iv^—Na1—O5	167.75 (4)
O7—Co1—O2	90.28 (4)	Co1—O1—H1A	119.7 (16)
O7—Co1—O2	90.28 (4)	Co1—O1—H1B	125.4 (16)
O7^i^—Co1—O2	89.73 (4)	H1A—O1—H1B	110 (2)
O1—Co1—O2^i^	86.86 (4)	P1—O2—Co1	128.01 (6)
O1^i^—Co1—O2^i^	93.14 (4)	P1—O3—Na1^v^	123.17 (6)
O7—Co1—O2^i^	89.72 (4)	P1—O3—H2	114.8 (19)
O7—Co1—O2^i^	89.72 (4)	Na1^v^—O3—H2	122.0 (19)
O7^i^—Co1—O2^i^	90.27 (4)	P1—O4—Na1^vi^	130.61 (6)
O2—Co1—O2^i^	180.00 (5)	P2—O5—Na1	120.44 (5)
O4—P1—O2	115.31 (6)	P2—O5—H5	112.6 (18)
O4—P1—O3	111.31 (6)	Na1—O5—H5	112.6 (18)
O2—P1—O3	105.44 (6)	P2—O6—Na1^vi^	121.18 (5)
O4—P1—C1	110.17 (6)	P2—O7—Co1	131.33 (6)
O2—P1—C1	109.98 (6)	Na1^vii^—O8—H6	111.4 (16)
O3—P1—C1	103.94 (6)	Na1^vii^—O8—H7	104.6 (17)
O7—P2—O6	115.45 (6)	H6—O8—H7	102 (2)
O7—P2—O6	115.45 (6)	P2—C1—P1	114.42 (7)
O7—P2—O5	106.61 (6)	P2—C1—H3	112.4 (12)
O7—P2—O5	106.61 (6)	P1—C1—H3	105.2 (12)
O6—P2—O5	109.49 (6)	P2—C1—H4	110.3 (12)
O7—P2—C1	108.36 (6)	P1—C1—H4	107.1 (12)
O7—P2—C1	108.36 (6)	H3—C1—H4	107.0 (17)
O6—P2—C1	109.14 (6)		
			
O4—P1—O2—Co1	−108.16 (8)	O6^iv^—Na1—O5—P2	140.99 (16)
O3—P1—O2—Co1	128.63 (7)	Na1^ii^—Na1—O5—P2	−161.48 (7)
C1—P1—O2—Co1	17.13 (9)	Na1^vii^—Na1—O5—P2	16.59 (7)
O1—Co1—O2—P1	111.59 (7)	O7—P2—O6—Na1^vi^	−74.24 (8)
O1^i^—Co1—O2—P1	−68.41 (7)	O7—P2—O6—Na1^vi^	−74.24 (8)
O7—Co1—O2—P1	24.95 (7)	O5—P2—O6—Na1^vi^	165.48 (5)
O7—Co1—O2—P1	24.95 (7)	C1—P2—O6—Na1^vi^	48.07 (7)
O7^i^—Co1—O2—P1	−155.05 (7)	O6—P2—O7—Co1	134.82 (7)
O4—P1—O3—Na1^v^	−126.78 (7)	O5—P2—O7—Co1	−103.34 (8)
O2—P1—O3—Na1^v^	−1.06 (8)	C1—P2—O7—Co1	12.09 (10)
C1—P1—O3—Na1^v^	114.66 (7)	O1—Co1—O7—P2	−136.95 (8)
O2—P1—O4—Na1^vi^	97.12 (8)	O1^i^—Co1—O7—P2	43.05 (8)
O3—P1—O4—Na1^vi^	−142.84 (7)	O2—Co1—O7—P2	−43.82 (8)
C1—P1—O4—Na1^vi^	−28.09 (9)	O2^i^—Co1—O7—P2	136.18 (8)
O7—P2—O5—Na1	−35.73 (8)	O7—P2—C1—P1	46.92 (9)
O7—P2—O5—Na1	−35.73 (8)	O7—P2—C1—P1	46.92 (9)
O6—P2—O5—Na1	89.81 (7)	O6—P2—C1—P1	−79.55 (8)
C1—P2—O5—Na1	−151.75 (6)	O5—P2—C1—P1	161.78 (7)
O8^ii^—Na1—O5—P2	−162.13 (6)	O4—P1—C1—P2	66.52 (9)
O3^iii^—Na1—O5—P2	81.72 (6)	O2—P1—C1—P2	−61.67 (9)
O4^iv^—Na1—O5—P2	−77.92 (7)	O3—P1—C1—P2	−174.14 (7)

Symmetry codes: (i) −*x*+1, −*y*+1, −*z*+1;  (ii) *x*, −*y*+3/2, *z*−1/2; (iii) −*x*+1, *y*+1/2, −*z*+1/2; (iv) −*x*, *y*+1/2, −*z*+1/2; (v) −*x*+1, *y*−1/2, −*z*+1/2; (vi) −*x*, *y*−1/2, −*z*+1/2; (vii) *x*, −*y*+3/2, *z*+1/2.

### Hydrogen-bond geometry (Å, °)

**Table:**

*D*—H···*A*	*D*—H	H···*A*	*D*···*A*	*D*—H···*A*
O8—H6···O7	0.86 (1)	1.88 (1)	2.7154 (15)	164 (2)
C1—H4···O1^i^	0.92 (2)	2.54 (2)	3.1449 (17)	123.9 (15)
C1—H3···O4^viii^	0.92 (2)	2.53 (2)	3.4366 (17)	168.4 (17)
O3—H2···O2^viii^	0.81 (3)	1.84 (3)	2.6394 (14)	176 (3)
O1—H1A···O6^ix^	0.83 (2)	1.98 (2)	2.8008 (14)	173 (2)
O8—H7···O1^ix^	0.83 (1)	2.57 (2)	3.2763 (15)	143 (2)
O1—H1B···O4^x^	0.83 (2)	1.84 (2)	2.6634 (15)	175 (2)
O5—H5···O6^xi^	0.82 (2)	1.81 (2)	2.6272 (14)	177 (3)

Symmetry codes:  (i) −*x*+1, −*y*+1, −*z*+1; (viii) *x*, −*y*+1/2, *z*−1/2; (ix) −*x*, −*y*+1, −*z*+1; (x) *x*, −*y*+1/2, *z*+1/2; (xi) −*x*, −*y*+1, −*z*.
